# Biogeography of intestinal mucus-associated microbiome: Depletion of genus *Pseudomonas* is associated with depressive-like behaviors in female cynomolgus macaques

**DOI:** 10.1016/j.jare.2024.05.013

**Published:** 2024-05-11

**Authors:** Xunmin Tan, Jing Wu, Hanping Zhang, Yifan Li, Yu Huang, Peng Zheng, Peng Xie

**Affiliations:** a NHC Key Laboratory of Diagnosis and Treatment on Brain Functional Disease, The First Affiliated Hospital of Chongqing Medical University, Chongqing, China; b Department of Neurology, The First Affiliated Hospital of Chongqing Medical University, Chongqing, China; c The Jin Feng Laboratory, Chongqing, China; d Institute for Brain Science and Disease, Chongqing Medical University, Chongqing, China

**Keywords:** Depression, Biogeography, Mucus-associated microbiota, Genus *Pseudomonas*, Lipid metabolism

## Abstract

**Introduction:**

Depression is a debilitating and poorly understood mental disorder. There is an urgency to explore new potential biological mechanisms of depression and the gut microbiota is a promising research area.

**Objectives:**

Our study was aim to understand regional heterogeneity and potential molecular mechanisms underlying depression induced by dysbiosis of mucus-associated microbiota.

**Methods:**

Here, we only selected female macaques because they are more likely to form a natural social hierarchy in a harem-like environment. Because high-ranking macaques rarely displayed depressive-like behaviors, we selected seven monkeys from high-ranking individuals as control group (HC) and the same number of low-ranking ones as depressive-like group (DL), which displayed significant depressive-like behaviors. Then, we collected mucus from the duodenum, jejunum, ileum, cecum and colon of DL and HC monkeys for shotgun metagenomic sequencing, to profile the biogeography of mucus-associated microbiota along duodenum to colon.

**Results:**

Compared with HC, DL macaques displayed noticeable depressive-like behaviors such as longer duration of huddle and sit alone behaviors (negative emotion behaviors), and fewer duration of locomotion, amicable and ingestion activities (positive emotion behaviors). Moreover, the alpha diversity index (Chao) could predict aforementioned depressive-like behaviors along duodenum to colon. Further, we identified that genus *Pseudomonas* was consistently decreased in DL group throughout the entire intestinal tract except for the jejunum. Specifically, there were 10, 18 and 28 decreased *Pseudomonas* spp. identified in ileum, cecum and colon, respectively. Moreover, a bacterial module mainly composed of *Pseudomonas* spp. was positively associated with three positive emotion behaviors. Functionally, *Pseudomonas*was mainly involved in microbiota derived lipid metabolisms such as PPAR signaling pathway, cholesterol metabolism, and fat digestion and absorption.

**Conclusion:**

Different regions of intestinal mucus-associated microbiota revealed that depletion of genus *Pseudomonas* is associated with depressive-like behaviors in female macaques, which might induce depressive phenotypes through regulating lipid metabolism.

## Highlights

• Chao index of intestinal mucus-associated microbiota was significantly associated with depressive-like behaviors in DL macaques.
• There were 10, 18 and 28 *Pseudomonas* spp. depleted in ileum, cecum and colon of DL, respectively.
• A bacterial module mainly composed of *Pseudomonas* spp. was positively associated with three positive emotion behaviors.
• *Pseudomonas* was mainly involved in microbiota derived lipid metabolisms such as PPAR signaling pathway, cholesterol metabolism, and fat digestion and absorption.

## Graphical abstract

**Figure:**
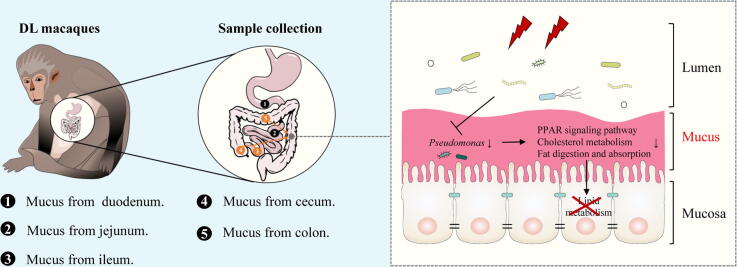

## Introduction

Major depressive disorder (MDD) is a common disease that has told severely on psychosocial function and diminished quality of life [1]. Moreover, depressive disorders have been expected to be the second leading cause of years lived with disability in China [2]. To date, a growing body of investigations suggest that MDD is related to a serial of biological, psychological and social factors [3], in which social stress is a major social risk factor [4]. To date, certain hypotheses concerning pathogenesis of MDD mainly devoted to neuroendocrine, neurogenesis and neuroplasticity [5], [6], but a definitive mechanism remains elusive. Therefore, it is of great value to detect new potential biological mechanisms of depression.

In recent years, it is a novel area of interest that gut microbiota mediates depression via the microbiota-gut-brain axis [7], [8]. Numerous experiments displayed dysbiosis of gut microbiota in depressed patients [9], [10] and animal models, including rodents [11] and non-human primates [12], [13]. Colonizing fecal suspension of MDD patients to germ-free mice or antibiotic treated mice leads to depressive-like behaviors [9], [14]. Aforementioned findings further suggested that disturbances of the gut microbiota may be a significant environmental factor contributing to depressive disorders. Nevertheless, previous studies mainly focused on luminal and mucosa-associated microbiota [15], [16], without paying attention to the changes of microbita in the mucus layer. The mucus layer is composed of mucus secreted by goblet cells, forming the chemical barrier of the intestinal barrier. Hence, characterization of microbial composition and function in the mucus layer is meaningful for getting a complete image of the gut microbiome. The physiology of the intestine varies dramatically along its length, forming unique environmental niches and creating a distinct microbiota biogeography that shapes the dynamics of microbial interactions with the host [17], [18], [19], [20]. Thus, it is of great significance to profile the biogeography of mucus-associated microbiota throughout the entire tract in female depressive-like macaques.

Previously, we have successfully constructed female depressive cynomolgus macaques models in an oppressive environment [12], [21]. Compared to rodent models of MDD, non-human primates displayed depressive-like behaviors due to more psychological and social stress. Secondly, non-human primates have a more similar genetic composition, brain anatomy, emotional patterns, and social behavior to humans than rodents [22], [23]. Moreover, cynomolgus macaques can compensate for the limited sampling of MDD patients. Thus, non-human primates provide a promising depression model for in-depth exploration of the pathogenesis of MDD. In addition, our previous studies have shown significant metagenomic, metabolomic and lipidomic variations in female DL macaques. To be specific, dysbiosis of gut microbiota led to depressive-like behaviors in female macaques through the lipid metabolism such as glycerophospholipid and 1,2-diacylglyceride metabolism [12], [13]. Hence, understanding biogeographic alterations in mucus-associated microbial composition and function of DL monkeys is a crucial supplement to previous investigations.

Here, we identified seven monkeys with depressive-like behaviors from low-ranking individuals and also selected seven control counterparts from high-ranking individuals, because high-ranking macaques rarely displayed depressive-like behaviors. Then, we initially characterized the biogeography of mucous bacterial composition and function along duodenum to colon using shotgun metagenome sequencing. Secondly, we conducted WGCNA analysis to explore key bacterial modules and investigate the relation of bacterial modules to depressive-like behaviors. At last, mapped to the KEGG database, most identified mucous bacteria in this study showed potential involvement in lipid metabolism.

## Materials and methods

### Ethics statement

All experiments complied with the recommendations in the “Guide for the Care and Use of Laboratory Animals” of the Institute of Neuroscience at Chongqing Medical University (approval number: 20100031). All procedures using nonhuman primates were conducted in accordance with the Guide for the Care and Use of Laboratory Animals (the 8th edition, NIH). All cynomolgus macaques (Macaca fascicularis) were housed and raised at the facility of Zhongke Experimental Animal Co., Ltd., Suzhou, P.R.C. Monkeys were raised in an animal room with humidity at 50 ± 5 %, temperature at 22 ± 1 °C, and a 12/12 light–dark cycle with lights on at 7:00 am.

### Social rank formation and behavioral observation

Here, we only selected female monkeys as our research subjects because most female macaques tend to live a harem-like lifestyle, which makes it easier for them to form a natural social hierarchy. The detailed experiment protocol and behavioral test were shown in our previous literatures [12], [21]. Briefly, we spent 2 years collecting over 4700 h of behavioral videos from 24 cages of macaques. First, three highly aggressive populations were screened out in line with the number of conflicts (227, 186 and 181, respectively). Next, social hierarchy evaluation of each population was carried out using DS analysis, a standard ranking method for calculating dominance ranks of members in a social group [24], [25]. Female monkeys in the high and low social ranks were selected for subsequent behavioral analysis. In view of a previously established behavioral observation system [12], [13], [26], termed the “free enclosures test”, five depressive-like activities were tested, containing huddle, sit alone, locomotion, amicable and ingestion. Eventually, we screened out seven monkeys displayed depressive-like behaviors as DL group, and seven healthy counterparts as HC group. To ensure the reliability of experimental model, all behavioral videos were independently analyzed by two experimenters unknown to experimental grouping.

### Subjects and sample collection

All monkeys were deeply anesthetized with pentobarbital, and incision of the abdominal wall was done to expose the entire gastrointestinal tract. We separated the duodenum, jejunum, ileum, cecum and colon, and longitudinally cut each segment of intestinal tract to expose the intestinal lumen. Next, we removed the intestinal contents and fixed the intestinal mucosa onto the anatomical disc using syringe needles, to eliminate wrinkles in the intestinal mucosa and relax the muscle layer. Then, we gently scraped the mucus from the surface of the mucosal layer, and immediately froze in dry ice. In total, 70 mucous samples were collected from five intestinal segments of 14 monkeys. All the samples were stored at −80 °C until further studies.

### DNA extraction, PCR amplification, and shotgun metagenomic sequencing

The shotgun metagenomic sequencing protocol was in accordance with our previous published study [9]. Briefly, we extracted microbial DNA from mucus using the E.Z.N.A® DNA kit (Omega Bio-Tek). The concentration and purity of microbial DNA were qualified and evaluated by Quantus Fluorometer (Picogreen) and NanoDrop 2000 spectrophotometer (Thermo Fisher Scientific). Then, the integrity of microbial DNA was assessed by 1 % agarose gel electrophoresis. Next, the qualified microbial DNA samples were fragmented using Covaris M220, and constructed PE library by NEXTFLEX ™ Rapid DNA-Seq Kit. Each library was sequenced on the Illumina HiSeq 4000 platform (Illumina Inc.) at Majorbio Bio-Pharm Technology Co., Ltd.

### Metagenomic analysis of mucous samples

First, raw-data optimization was carried out by splitting, quality shearing, and removing contamination. Host reads were filtered based on the sequence alignments with the Bayesian model averaging using Burrows-Wheeler Alignment Tool version 0.7.17 (https://bio-bwa.sourceforge.net/) [12]. Low-quality sequences were removed using the Sickle algorithm version 1.33 (https://github.com/najoshi/sickl) [27]. The remaining high-quality sequences were assembled using SOAPaligner soap2.21release (https://github.com/ShujiaHuang/SOAPaligner) [28] to assess the gene abundance in each sample. Next, the nonredundant gene sets were aligned against the NR database (nr_20200604, https://ftp.ncbi.nlm.nih.gov/blast/db/FASTA/) for taxonomic annotations using DIAMOND software version 0.8.35 (https://ab.inf.uni-tuebingen.de/software/diamond/) [29]. Then, the sum of gene abundance corresponding to each taxa was equal to the abundance profile of the taxa. Similarly, Kyoto Encyclopedia of Genes and Genomes (KEGG) annotation was performed with BLASTP against the KEGG database version 94.2 (https://www.genome.jp/kegg) using DIAMOND software.

Alpha diversity was used to assess mucus-associated microbial diversity, including microbial richness index (Chao) and diversity index (Shannon) [9]. Beta diversity was compared by principal coordinate analysis (PCoA) based on Bray-Curtis distance. PERMANOVA (permutational multivariate analysis of variance) was used to quantify the significance of β-diversity between DL and HC group. The key discriminative mucus-associated bacterial taxa and function were identified by Linear discriminant analysis Effective Size (LEfSe) [30]. The conditions for distinguishing differences: linear discriminant analysis (LDA) values > 2.0 and p values < 0.05.

### Weighted gene co-expression network analysis (WGCNA)

In this study, WGCNA was performed to determine bacterial modules related to depressive-like phenotypes [31]. WGCNA was conducted based on official tutorials (https://horvath.genetics.ucla.edu) using WGCNA package (v.1.72–1). Picking a soft thresholding parameter (β) value is the key to construct a scale-free network topology. Seven was the minimum β value satisfying R2 > 0.9, so it was chosen as the best β value in this study. Moreover, we used a ‘one-step network construction’ for microbial network topology. Pearson correlation was implemented to evaluate associations between depressive-like phenotypes and modules. Finally, significant correlations (|coefficient| > 0.40 and P < 0.05) were identified as behavior-associated modules.

### Statistical analysis

We conducted statistical analysis using SPSS (version 21.0) and R studio (version 2023.03.0), and visualized experimental results using GraphPad Prism (version 9.0) and Cytoscape (version 3.10.0). Two-sided student’s *t* test was performed to identify differences between DL and HC group in continuous variables such as behavioral phenotypes and alpha diversity indices. Correlations between depressive-like phenotypes and Chao index or abundance of genus *Pseudomonas* were analyzed by Spearman's correlation analysis. LEfSe analysis was used to identify discriminative taxa and function based on LDA > 2 and P value < 0.05. Statistical significance was set at <0.05.

## Results

### Cynomolgus macaques from a low social rank population display depressive-like behaviors

Detailed information for construction and evaluation of social stress-associated depressed cynomolgus monkeys were previously reported [21]. Briefly, we identified seven monkeys with depressive-like behaviors from low-ranking individuals and also selected seven healthy controls from high-ranking individuals. Luminal-associated microbiota is unable to completely grasp the overall picture of gut microbiome. Thus, investigation of microbial composition in the mucus layer is an important supplement for getting a complete image of the gut microbiome.

**Fig. 1:**
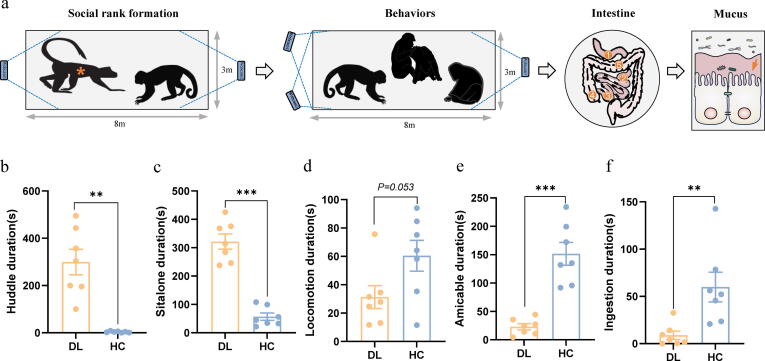
Schematic of the experimental design and behavioral results. (a) Flow diagram of the experimental procedure and the sample collection. ①duodenum, ②jejunum, ③ileum, ④cecum, ⑤colon. (b-f) Comparison of duration of huddle, sit alone, locomotion, amicable and ingestion activities between DL and HC groups. (DL, n = 7; HC, n = 7; *P < 0.05, **P < 0.01, ***P < 0.001, two-sided Student’s *t* test; bars show Mean ± SEM).

In view of a previously established behavioral testing methods [21], [32], we evaluated five depressive-like behavior indices, including huddle, sit alone, locomotion, amicable and ingestion. Compared with HC group, DL monkeys displayed longer duration of huddle (*P = 0.002,* Fig. 1b) and sit alone (*P < 0.001,* Fig. 1c) behaviors, and fewer duration of locomotion (*P = 0.053,* Fig. 1d), amicable (*P < 0.001,* Fig. 1e) and ingestion (*P = 0.009,* Fig. 1f) activities (Table S1). Moreover, there was no significant difference in age (HC: 18.86 ± 1.95, DL: 19.00 ± 2.77; *P = 0.913*) between DL and HC groups. But the weight of depressive-like macaques was significantly decreased relative to healthy counterparts (HC: 5.68 ± 0.91, DL: 3.34 ± 0.35; *P < 0.001*) (Fig.S1). These findings indicated that certain cynomolgus macaques from a low social rank population showed depressive-like behaviors.

### Biogeographic changes of mucous microbial diversity in DL monkeys

To examine the biogeography of mucus-associated microbiota along duodenum to colon, we collected mucus from the duodenum, jejunum, ileum, cecum and colon of DL and HC monkeys for metagenomic sequencing (Fig. 1a). However, two samples from the jejunum and ileum of DL monkeys were not sequenced due to inadequated sample volumes. Finally, 68 mucous samples were used to carry out whole-genome shotgun sequencing.

**Fig. 2:**
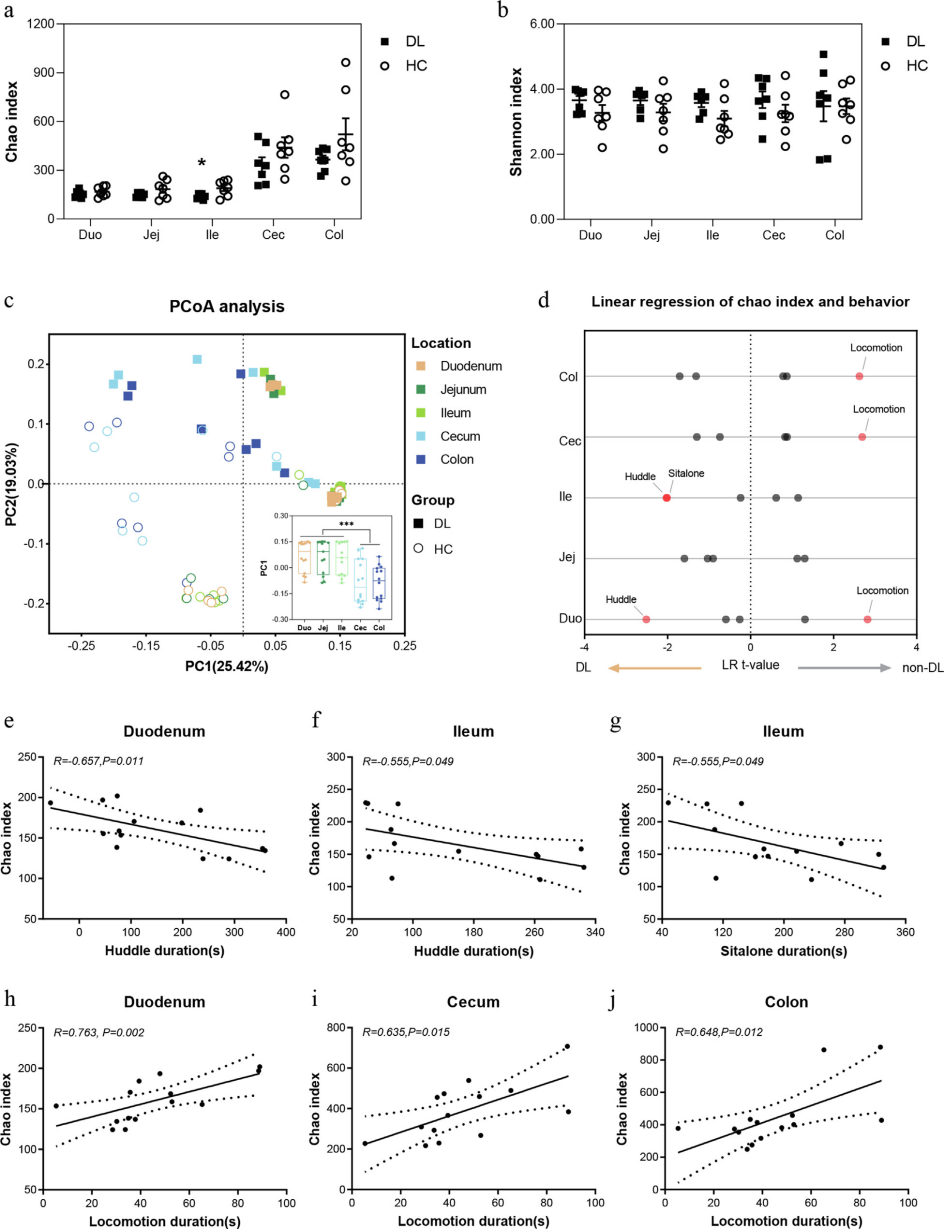
The biogeographical features of mucous microbiome diversity in DL versus HC. (a-b) Comparison of the alpha diversity was measured with Chao and Shannon indices at the species level for DL and HC group using Student’s *t* test; filled squares: DL group; empty circles: HC group. Mean ± SEM, dots represent individual samples. (c) Beta diversity was analyzed using PCoA analysis based on Bray-Curtis distance (two-way PERMANOVA, location: R2 = 0.78, P < 0.001; group: R2 = 1.98, P < 0.001). (d) Linear regression relationship between chao index and behaviors throughout the gut. (e–j) Scatter diagram showing the correlation of depressive-like behaviors with Chao index along the intestinal tract.

We obtained an average of 98,863,756 high-quality reads per sample from the metagenomic sequencing across all 68 mucus samples with an average length of 149.86 base pairs (bp). Initially, α-diversity analysis showed that except for Chao index in the ileum, which was significantly decreased in DL group (*P = 0.029*), no significant differences were found in other parts of the intestine between DL and HC groups (Fig. 2a-b). PCoA analysis revealed a robust clustering based on the longitudinal location and on the depression phenotype (Fig. 2c). Longitudinally, distribution of PC1 in small intestine was remarkably different from that in large intestine, while there was no significant difference among various parts of the small intestine or large intestine (Fig. 2c). In addition, we identified a significant difference in beta diversity between DL and HC groups in the small intestine, but not in the large intestine (Fig. S2).

### Αlpha-diversity predicts depressive-like behaviors

To illuminate new potential pathophysiologica mechanisms of depression, we investigated the relation of mucous microbial diversity with depressive-like behaviors in female cynomolgus macaques. As shown in Fig. 2d, Chao index could predict duration of huddle, sit alone and locomotion via linear regression analysis after adjustment for body weight and age. Further spearman correlation analysis revealed that Chao index was negatively associated with huddle and sit alone duration in duodenum (Huddle: *R = −0.657*, *P value = 0.011,* Fig. 2e) and ileum (Huddle: *R = −0.555*, *P value = 0.049,* Fig. 2f; Sit alone: *R = −0.555*, *P value = 0.049,* Fig. 2g), and positively associated with locomotion duration in duodenum (*R = 0.763*, *P value = 0.002,* Fig. 2h), cecum (*R = 0.635*, *P value = 0.015,* Fig. 2i) and colon (*R = 0.648*, *P value = 0.012,* Fig. 2j). Consistent with previous study, these findings suggested that rich α-diversity may represent a “good” health status.

### Microbiome composition differs along distinct intestinal regions

**Fig. 3:**
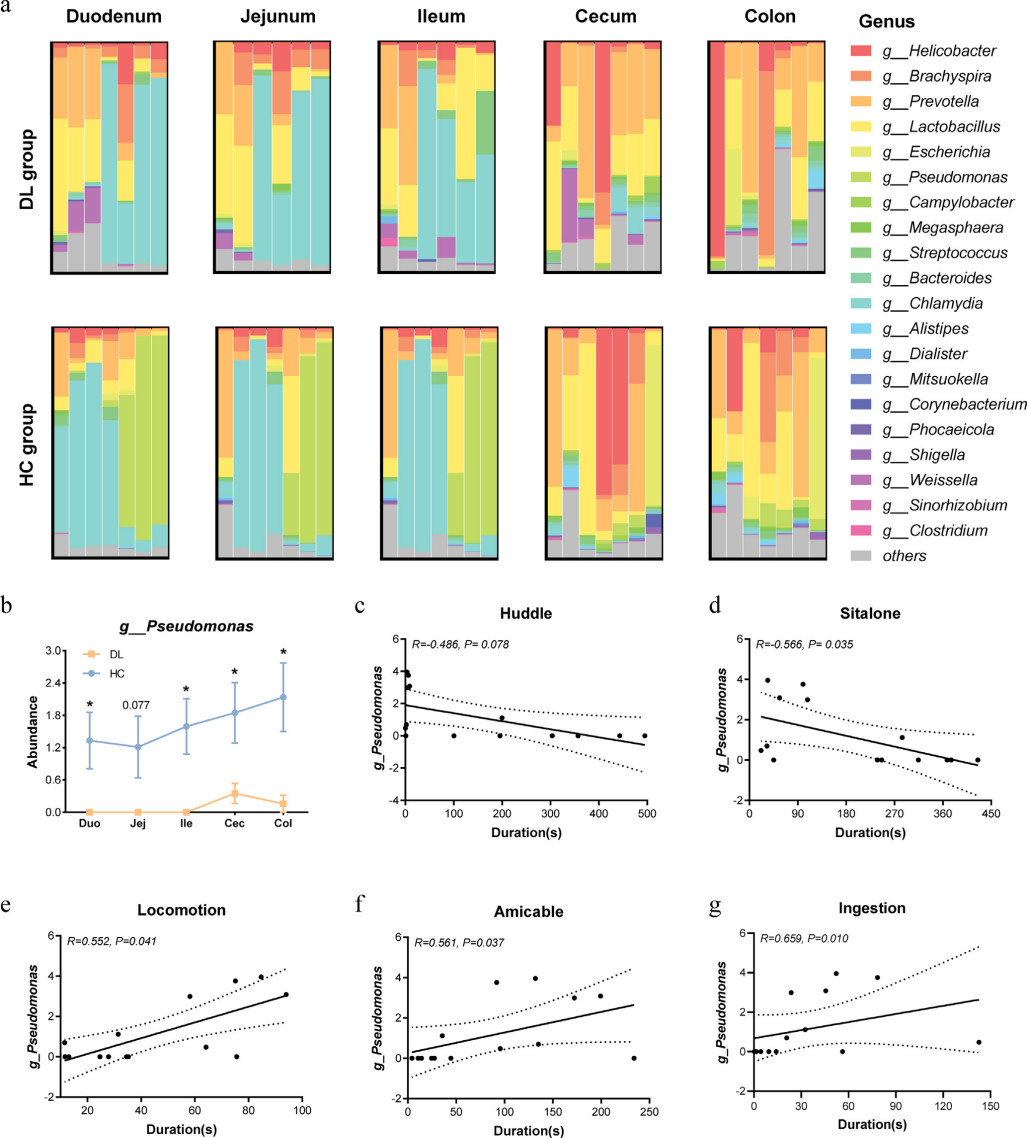
Biogeographical features of mucus-associated microbial composition along duodenum to colon. (a) Taxonomic distributions of mucus-associated bacterial composition at genus level. (b) Comparison of the abundance of genus *Pseudomonas* between DL and HC group. Data were presented as Mean ± SEM (*P < 0.05, two-sided Student’s *t* test). (c–g) Scatter diagram showing the correlation of depressive-like behaviors with the abundance of genus *Pseudomonas* in the colon.

To explore the dominant bacteria in mucus layer of DL monkeys, we summarized mucus-associated bacterial genera and compared the abundance of the main genera. Here, the mucous microbiome was mainly composed of 20 genera (Fig. 3a). For example, the dominant bacterial genera in small intestine were genus *Chlamydia*, *Pseudomonas*, *Prevotella* and *Lactobacillus*, whereas genus *Helicobacter*, *Brachyspira*, *Prevotella* and *Lactobacillus* were enriched in large intestine. To further quantified the bacterial differences, we compared the abundance of top 10 dominant genera in the mucus of cynomolgus macaques along the intestinal tract (Table S2 and Fig.S3). We found that genus *Pseudomonas* was significantly decreased in DL group throughout the entire intestinal tract except for the jejunum (Fig. 3b). In the colon, genus *Pseudomonas* was negatively associated with sit alone but not huddle (huddle: *R = −0.486*, *P value = 0.078,* Fig. 3c; sit alone: *R = −0.566*, *P value = 0.035,* Fig. 3d) duration, and positively associated with three positive emotion behaviors (locomotion: *R = 0.552*, *P value = 0.041,* Fig. 3e; amicable: *R = 0.561*, *P value = 0.037,* Fig. 3f; ingestion: *R = 0.659*, *P value = 0.010*, Fig. 3g; respectively).

### Discriminative bacterial species between DL and HC group

**Fig. 4:**
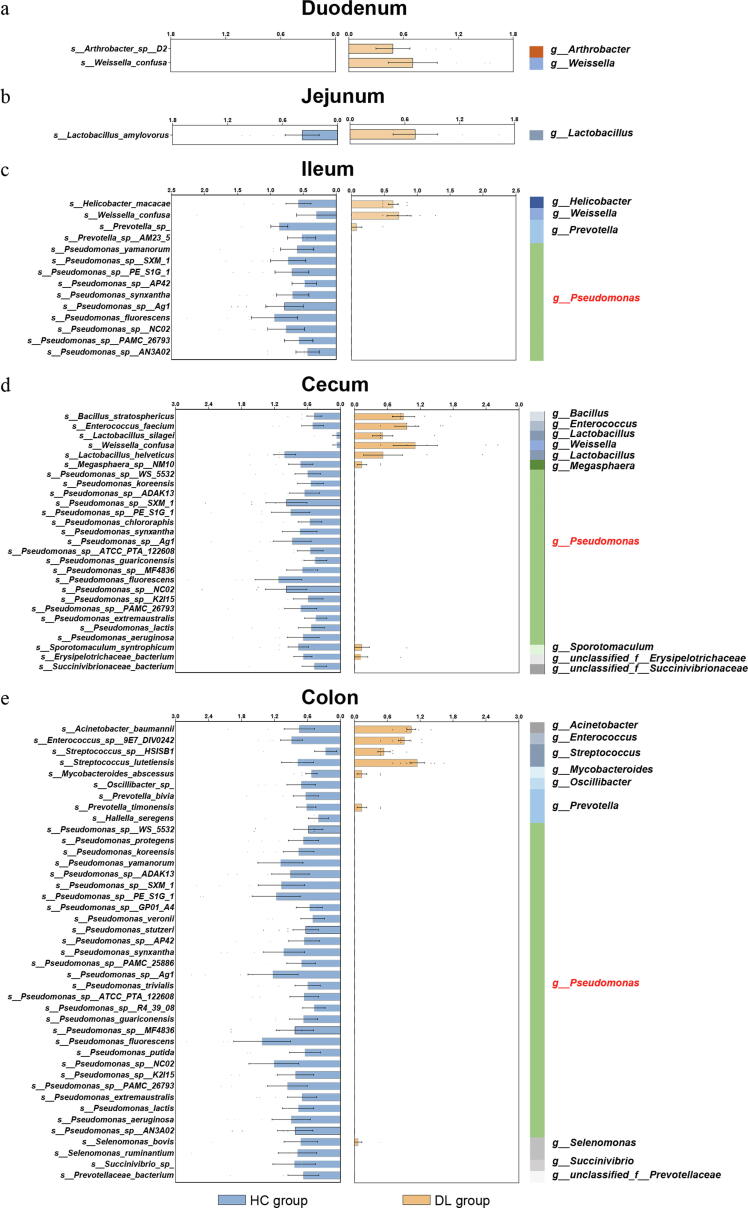
The discriminative bacterial species between DL and HC groups. (a) Two bacterial species were increased in DL group compared to HC in duodenum. (b) One bacterial species, assigned to genus *Lactobacillus*, was enriched in jejunum of the DL group. (c) 14 bacterial species were differentially expressed in DL and HC groups in ileum. (d) 27 bacterial species differed between the two groups in cecum. (e) 41 bacterial species responsible for discriminating DL and HC groups were identified in colon. The genus classification of each species is displayed on the left.

Next, we conducted LEfSe analysis to further identify the dominant differential bacteria at species level. Interestingly, differential bacterial species mainly appeared in ileum, cecum and colon, but not in duodenum and jejunum. In total, we identified 14, 27 and 41 differential species in ileum, cecum and colon, respectively (Table S3). However, only 2 and 1 differential bacterial species were detected in the duodenum and jejunum (Fig. 4a-b). In addition, most of these differential species were consistently downregulated in the ileum, cecum and colon of DL monkeys. Compared with HC, 12 (12/14, 85.7 %) species were decreased in the ileum of DL group, in which 10 species belonged to the genus *Pseudomonas* (Fig. 4c). Additionally, there were 23 (23/27, 85.2 %; Fig. 4d) and 37 (37/41, 90.2 %; Fig. 4e) species depleting in the cecum and colon of DL group. Similarly, these bacteria mainly belonged to the genus *Pseudomonas*. These findings suggested that the mucous bacteria of DL monkeys were characterized by the reduction of genus *Pseudomonas* in the lower part of intestine tract.

### Perturbed mucous microbial modules in DL monkeys

**Fig. 5:**
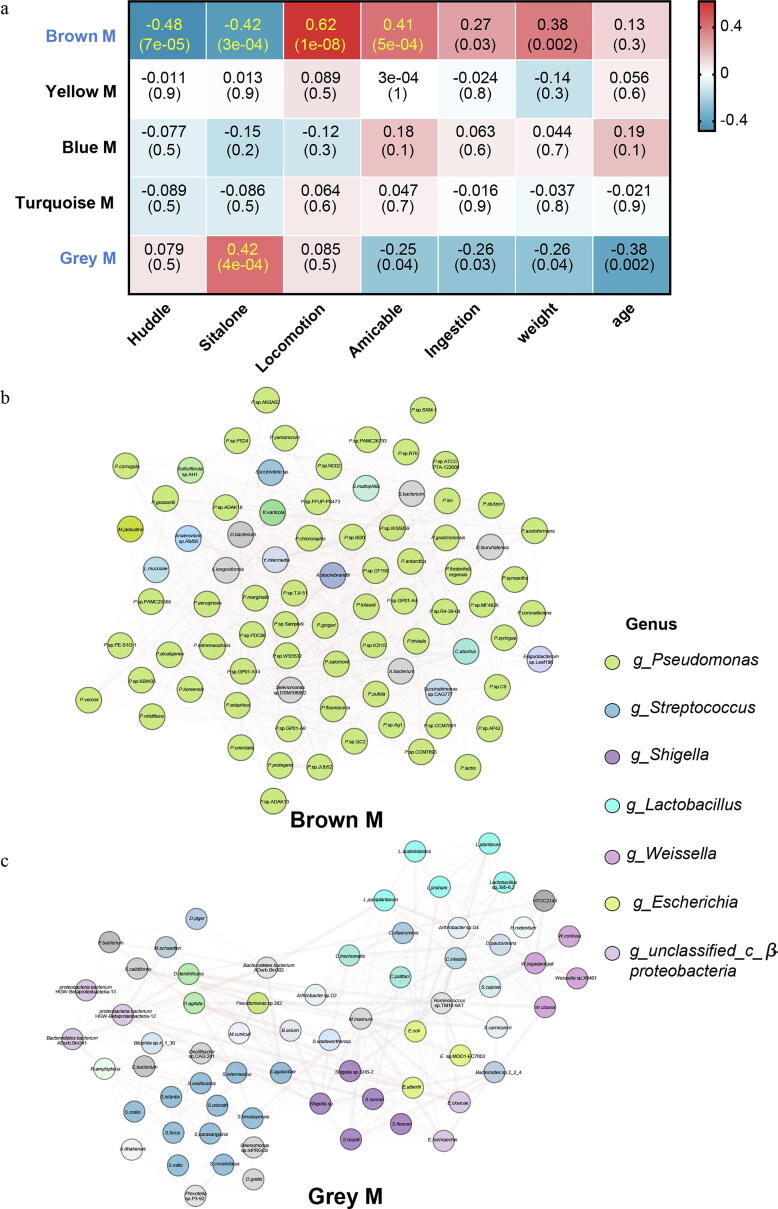
The mucous bacterial species were associated with depressive-like phenotypes. (a) Heat map of correlation coefficients between bacterial modules and behaviors. The blue and red color indicated a negative or positive correlation between each modules and behaviors, respectively. The Pearson’s correlation coefficients and P values were shown within the squares. (b-c) Network diagrams of differential bacterial species in brown and grey modules. Circle colors indicated the taxonomic assignment of each species. (For interpretation of the references to color in this figure legend, the reader is referred to the web version of this article.)

Here, we carried out WGCNA analysis to identify DL-associated bacterial modules without intestinal segment separation. This analysis yield 5 coexpression bacterial modules from 594 different species after filtering missing values (Table S4a). Two metagenomic modules (M-brown, M-grey) were significantly associated with DL behaviors (Fig. 5a). Specifically, the brown module was negatively associated with duration of huddle (*R = −0.48, P = 7e−05*) and sit alone (*R = −0.42, P = 3e−04*), and positively associated with duration of locomotion (*R = 0.62, P = 1e−08*), amicable (*R = 0.41, P = 5e−04*) and ingestion (*R = 0.27, P = 0.03*). On the countrary, the grey module was positively associated with duration of sit alone (*R = 0.42, P = 4e−04*), and negatively associated with duration of amicable (*R = −0.25, P = 0.04*) and ingestion (*R = −0.26, P = 0.03*). These results indicated that the brown module maybe an antidepressant module, but the grey module maybe a depressive module.

The brown module clustered 81 bacterial species, 63 of which belonged to genus *Pseudomonas* (Fig. 5b and Table S4b)*.* However, the grey module consisted of 66 bacterial species, which belonged to diverse genera at genus level. Of these, genus *Streptococcus* (11/66, 16.7 %) contributed mostly to the grey module, followed by genus *Lactobacillus* and *Shigella* (5/66, 7.6 %) (Fig. 5c and Table S4c).

### Significant variations exhibit in microbial functions related to lipid metabolism

**Fig. 6:**
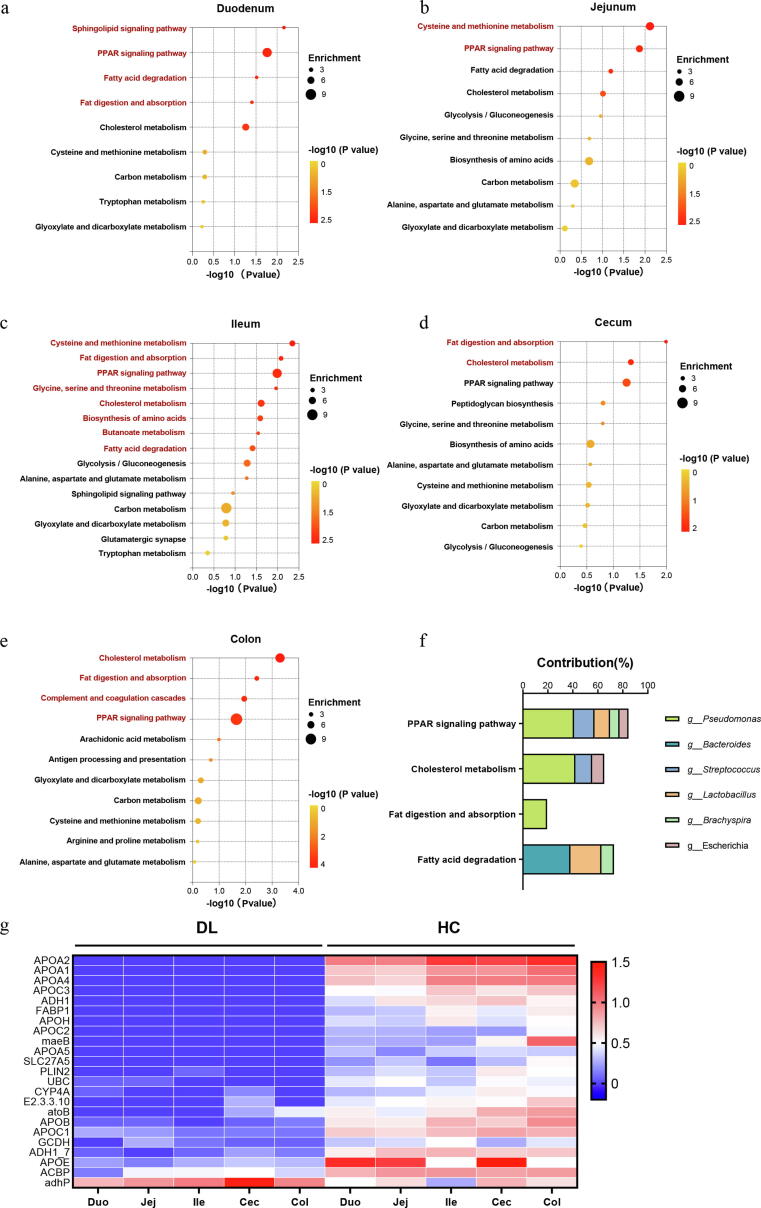
Key metabolic pathways related to mucus-associated microbial genes. Key metabolic pathways related to microbial gene in duodenum (a), jejunum (b), ileum (c), cecum (d) and colon (e). Circle size and color in each plot indicated enrichment of microbial genes and levels of significance. Red labels indicated significantly downregulated metabolic pathways in DL group. (f) The contribution of the most abundant genera to key metabolic pathways significantly changed in multiple intestinal segments. (g) Heat map of differentially expressed genes involved in lipid metabolism. Red and blue colors indicated up- and down-regulated gene expression, respectively. (For interpretation of the references to color in this figure legend, the reader is referred to the web version of this article.)

Using LEfSe analysis, we totally screened out 779 discriminative KOs between DL and HC groups along duodenum to colon (Table S5). These KOs were involved in various metabolic pathways, especially the lipid metabolism. Among them, four metabolic pathways including peroxisome proliferator-activated receptors (PPAR) signaling pathway, cholesterol metabolism, fat digestion and absorption and fatty acid degradation, were significantly depleted in multiple intestinal segments of DL monkeys (Fig. 6a–e and Table S6a–e). Based on the reads number of genera and functions in each sample, distance-based redundancy analysis (db-RDA) was performed to identify the bacterial contribution to specific function. Consequently, we found that genus *Pseudomonas* was mainly contributed to three bacterial functions, including PPAR signaling pathway (Contribution 41.0 %, *P value* 0.002), cholesterol metabolism (Contribution 42.0 %, P *value* 0.002) and fat digestion and absorption (Contribution 19.4 %, *P value* 0.048). Metagenomic function of fatty acid degradation was mainly contributed by genus *Bacteroides* (Contribution 38.1 %, *P value* 0.002) and genus *Lactobacillus* (Contribution 24.8 %, *P value* 0.002) (Fig. 6f and Table S6f).

Compared with HC group, there was a remarkable depletion in genes encoding enzymes that involved in PPAR signaling pathway, cholesterol metabolism, fat digestion and absorption, and fatty acid degradation (Fig. 6g). PPARs belong to ligand induced nuclear receptors, which consist of three subtypes including PPARα, PPARβ/δ and PPARγ [33]. Activated PPARs heterodimerize with retinoid X receptor (RXR) forming heterodimers that regulate the expression of genes involved in lipid metabolism, maintenance of metabolic homeostasis and inflammation. In this study, there were substantial reductions in lipid transport genes such as *Apo A2*, *Apo A5* and *Apo C3*, fatty acid transport genes such as *ACBP* and *FABP1*, fatty acid oxidation genes such as *CYP4A1* (Fig.S4). Taken together, these data demonstrated that microbial lipid metabolism disturbance may be an important pathway for mucous bacteria to regulate depressive-like behaviors.

## Discussion

In this study, we firstly depicted the biogeography of mucus-associated microbiota along duodenum to colon and identified a group of reduced microflora involved in lipid metabolism in female DL macaques. First of all, Chao index was significantly associated with depressive-like behaviors in both small and large intestine. At genus level, *Pseudomonas* was consistently decreased in DL group throughout the entire intestinal tract except for the jejunum. Specifically, we identified 10, 18 and 28 *Pseudomonas* spp. decreased in ileum, cecum and colon, respectively. A bacterial module, which was mainly composed of species from genus *Pseudomonas,* was positively associated with three positive emotion behaviors. In addition, *Pseudomonas* was mainly involved in microbiota derived lipid metabolisms such as PPAR signaling pathway, cholesterol metabolism, and fat digestion and absorption.

Previously, a substantial amount of clinical researches have reported drastical disturbance of gut microbiota in patients with MDD [9], [10], [34]. However, these findings were not absolutely consistent as a result of demographic diversity among different cohorts and differences in sequencing and analytical methods. In this study, these female macaques have the same living environment, which avoided those confounding factors such as heterogeneity of diet in human, and better mimiced the human experience than rodents. Further, previous studies mainly focused on variations in gut microbiome of the whole luminal contents [11], [12], with little attention paid to changes of gut microbiome in mucus layer. Physiologically, the gut epithelial cells and commensal bacteria secrete some adhesive substances that cover the surface of the mucosa to construct chemical and physical barriers, segregating the intestinal microbiota from epithelial cells and indirectly interacting with each other [35]. Herein, we explored the biogeography of mucus-associate microbiota along duodenum to colon, contributing to identify potential gut bacteria that elicit host-protective metabolism by direct interaction with the underlying epithelium. An understanding of the intimate interactions between the gut microbiota and epithelial cells that are crucial for the maintenance of intestinal homeostasis might promote advances in diagnostic and therapeutic approaches for depression.

Recently, Bosch et al. found from a large sample of clinical investigation that diversity of the gut microbiota could predict depressive symptom levels in MDD patients without discrepancy between ethnic groups [36]. Here, inspection of regression coefficients demonstrated alpha-diversity predicted depressive-like phenotypes in female macaques after adjusting for weight and age. For example, alpha-diversity (Chao index) was positively related to positive emotion behaviors, whereas negatively correlated with negative emotion behaviors. Our results indicated that α-diversity of intestinal microbiota exhibited consistent variations both across species and over different dimensions of the intestine including the intestinal lumen and mucus. Taken together, then, these findings were in line with the concept of α-diversity as a generic biomarker of health and disease (including depression) [37].

The second major finding in this study was the depletion of *Pseudomonas* in female DL macaques. DL monkeys in our study suffered chronic social stress from dominant individuals. Numerous studies have reported that stress can result in gut microbiota disorders [38], [39], [40]. *Pseudomonas* belongs to the family *Pseudomonadaceae* and phylum *Proteobacteria/Pseudomonadota*, which is the common member of healthy gastrointestinal tract [41], [42]. Therefore, we inferred that depletion of genus *Pseudomonas* may be a result of stress-induced disruption of gut microbiota. However, our previous published research has revealed that decreased lumina-associated bacteria in DL monkeys were mainly genera *Gemella* and *Streptococcus* [13]. These variant results may indicate that not all the changed luminal bacteria directly interact with intestinal epithelial cells. Several families belonging to the phylum Proteobacteria/Pseudomonadota were reduced in patients with MDD and related to lipoprotein disorders in blood [43]. Extracts from *Pseudomonas* spp. have protective effects against anxiety and depression-like behaviors through antioxidant activities in mice [44], [45]. *Pseudomonas* also have the ability to control hyperuricemia by the promotion of purine and uric acid catabolism [42], [46]. Overall, *Pseudomonas* may play protective roles in maintaining host health through the exclusive strain-specifc metabolic effects, suggesting the potential biological beneficial effects of traditional harmful bacteria. Future studies should be conducted to identify specific *Pseudomonas* spp. strains and clarify the underlying antidepressant mechanisms, providing new potential intervention targets for the treatment of depression.

As wellknown, lipids account for 50 % of brain dry mass [47]. Lipids play a crucial role in maintaining fundamental neurobiological functions including neuronal membrane formation and homeostasis maintenance, as well as synaptic transmission and trafficking [48]. In this study, we found that *Pseudomonas* was mainly involved in microbiota derived lipid metabolisms such as PPAR signaling pathway, cholesterol metabolism, and fat digestion and absorption. Our previous study has demonstrated that altered lipid metabolism occurr in gut–brain axis of depressive-like macaques. These are characterized by consistent enrichment of peripheral and central fatty acyl, sphingolipid and glycerophospholipid metabolism [32]. Numerous studies have reported that depression is associated with peripheral and central disorders of lipid metabolism. Moreover, the gut microbiota has been proven to be a major environmental factor impacting circulating lipids [49], [50], [51]. Collectively, we inferred that mucus-associated microbiota may contribute to the onset of DL behaviors through modulating lipid metabolism. Further, the gut microbiota can directly activate the activity of PPARγ by repressing the expression of lncRNA Snhg9 [52]. And gut microbiota-derived inosine, endocannabinoids (eCBs) and short chain fatty acids (SCFAs) can also activate the PPAR signaling pathway [53], [54]. In addition, neuron-specific PPARγ deficiency in mPFC is sufficient to induce depressive-like behaviors, which can be recovered by overexpressing PPARγ [55]. Herein, PPAR signaling pathway may be a key node in gut microbiota mediated depression.

We acknowledge the following strengths and limitations of our study. A major strength of this study is that to our knowledge, we first describe the biogeography of mucus-associated microbiota along duodenum to colon in non-human primates and identify a group of reduced microbiota mediated depression by regulating lipid metabolism. Potential limitations and future work for this study: (i) due to the rarity of cynomolgus macaques and animal ethics, the limited sample size may decrease the reliability of relevant analysis such as WGCNA, which may be affected; (ii) due to the harem-like lifestyle of female cynomolgus macaques, social hierarchy are more likely to form in female Macaca populations. Therefore, this study only chooses female macaques as research subjects, which may lead to sex bias; (iii) due to the limited amount of intestinal mucus, microbiota functional changes have not been fully validated, future studies should collect fresh mucus samples for the detection of microbita derived metabolites such as SCFAs; (iv) our findings are unable to prove the causal roles between depletion of genus *Pseudomonas* and depressive-like behaviors in DL monkeys. Future studies are needed to isolate and culture *Pseudomonas* spp. in vitro, and colonize them to germ free or antibiotic intervention animals to observe whether the colonized animals displayed antidepressant phenotype and activated PPAR signaling pathway.

## Conclusions

This study initially profiled the biogeography of mucous bacterial communities in female cynomolgus macaques with depressive-like behaviors by means of metagenomic sequencing. Further, we found that *Pseudomonas* was significantly depleted in the mucus of ileum, cecum and colon of DL macaques, which was mainly involved in lipid metabolism. Overall, our results shed new lights on understanding the relationship between gut microbiota and depression.

## Compliance with ethics requirements

All experiments complied with the recommendations in the “Guide for the Care and Use of Laboratory Animals” of the Institute of Neuroscience at Chongqing Medical University (approval number: 20100031).

## Declaration of competing interest

The authors declare that they have no known competing financial interests or personal relationships that could have appeared to influence the work reported in this paper.
