# Supplementary material for: Biogeography of intestinal mucus-associated microbiome: Depletion of genus Pseudomonas is associated with depressive-like behaviors in female cynomolgus macaques
Source: J Adv Res. 2024 May 11;70:393–404. doi: 10.1016/j.jare.2024.05.013 (PMC11976423; doi:10.1016/j.jare.2024.05.013)
Supplement: Supplementary Data 1 [file mmc1.docx]

**Biogeography of intestinal mucus-associated microbiome:** **depletion of genus *Pseudomonas* is associated with depressive-like behaviors** **in female cynomolgus macaques**

Xunmin Tan, Jing Wu, Hanping Zhang, Yifan Li, Yu Huang, Peng Zheng, Peng Xie

***Supplemental Information***

**Supplemental Figures**


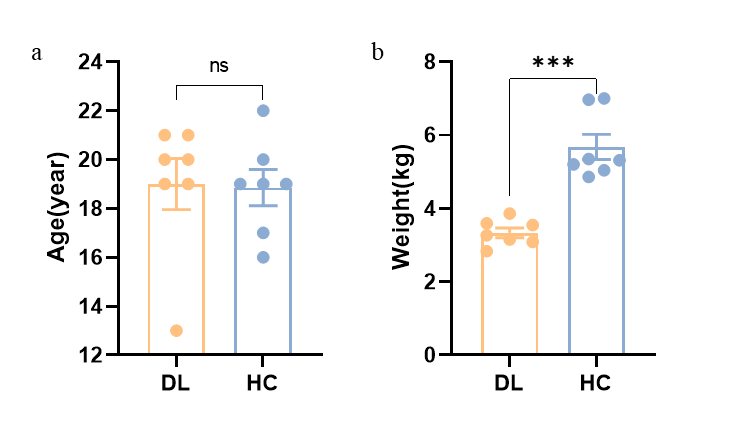


**Fig.S1. Information about the age and weight of female macaques.** (a) No significant difference was found in age between DL and HC macaques (HC: 18.86 ± 1.95, DL: 19.00 ± 2.77; *P=0.913*). (b) Compared with HC group, the weight of DL group was significantly decreased (HC: 5.68 ± 0.91, DL: 3.34 ± 0.35; *P<0.001*).

**
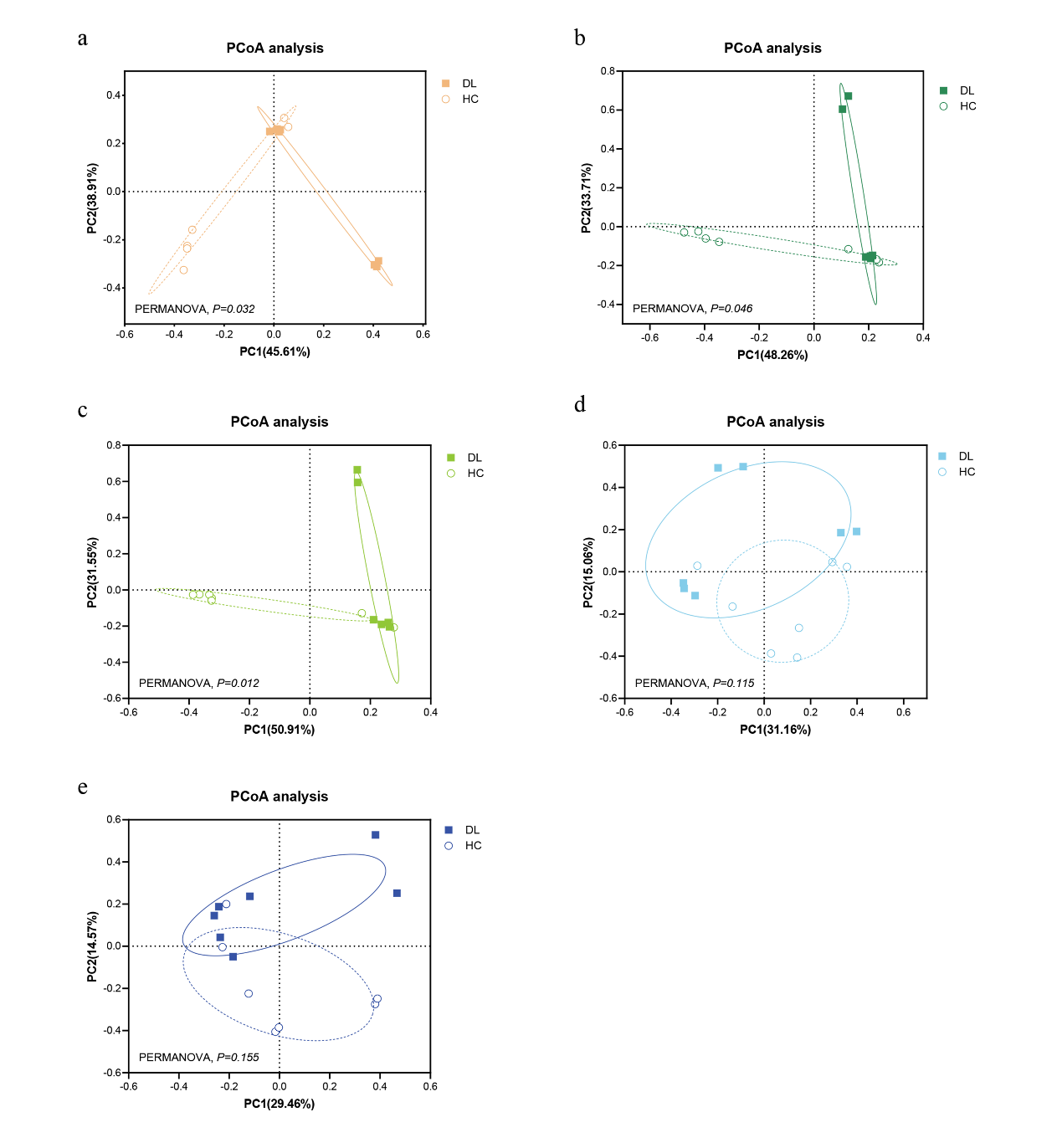
**

**Fig.S2. Specific comparison of the beta diversity between DL and HC.** (a-c) Bacterial signatures between the two groups were significantly different in the duodenum (PERMANOVA, *P* = 0.032), jejunum (PERMANOVA, *P* = 0.046) and ileum (PERMANOVA, *P* = 0.012). (d-e) Overall bacterial signatures of the DL group were not significantly discriminated from the HC group in cecum (PERMANOVA, *P* = 0. 115) and colon (PERMANOVA, *P* = 0. 155).

**
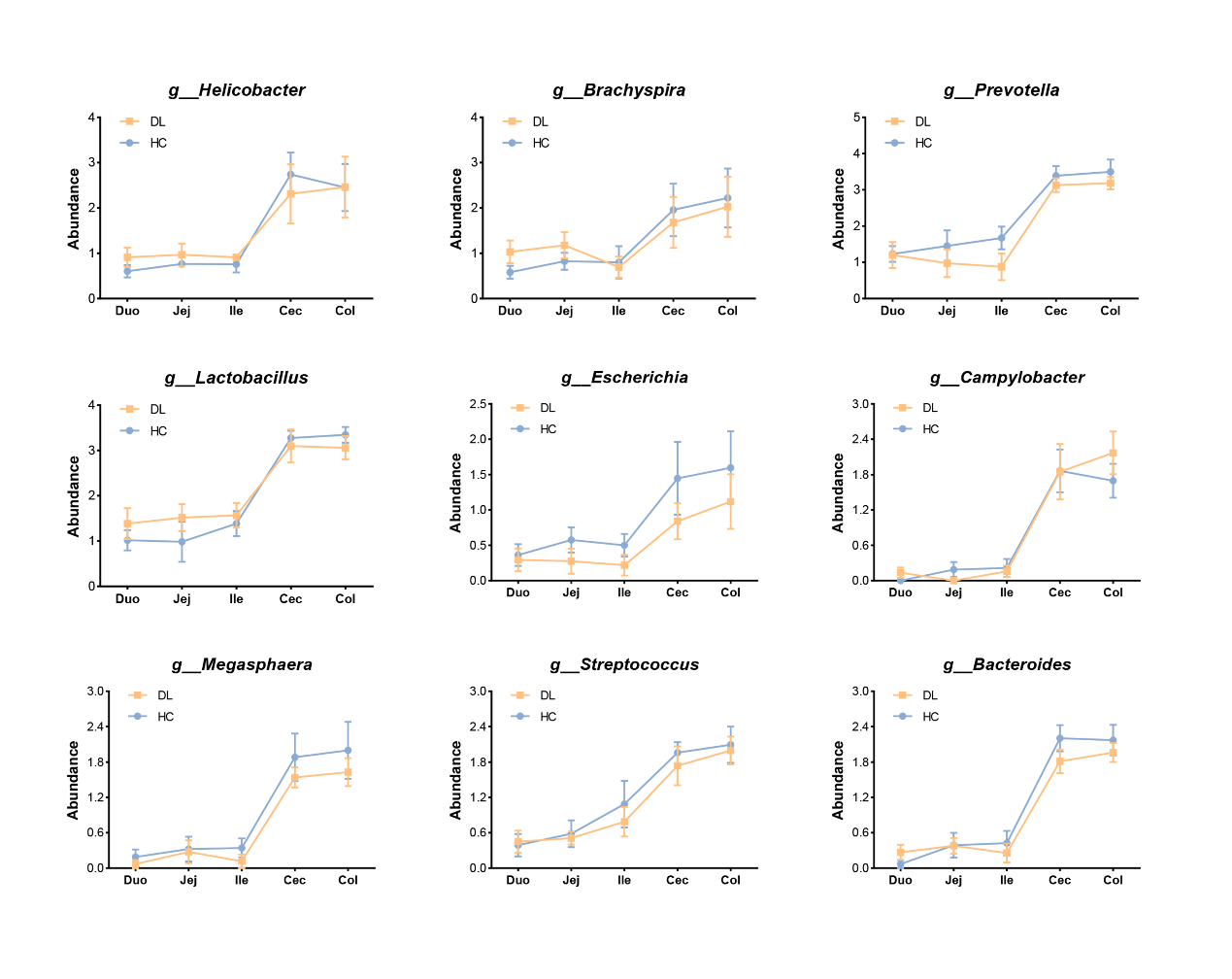
**

**Fig.S3. Comparison of the abundance of several most abundant genera in depressive-like (DL) and healthy control (HC) macaques.** There was no significant difference in above mentioned genera between DL and HC group. Data were presented as Mean ± SEM (two-sided Student’s t test).


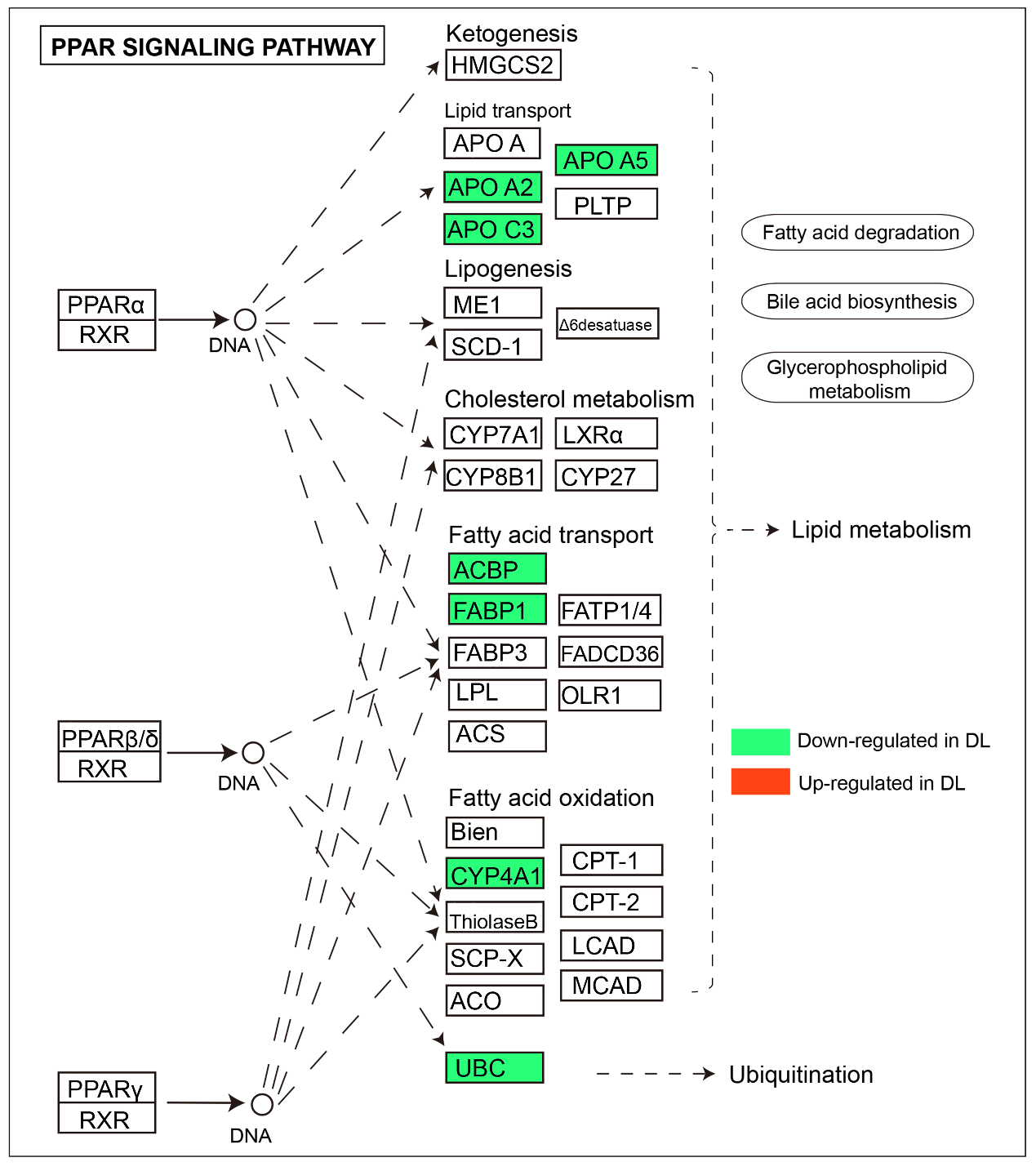
**Fig.S4. Pathway diagram of PPAR signaling pathway (map03320).** This pathway information was referred to the Kyoto Encyclopedia of Genes (KEGG). Red and green colors indicated up-regulated and down-regulated gene expression in DL group, respectively.

**Supplemental Tables**

**Table S1.** Behavioral results and basic information of enrolled female cynomolgus macaques.

**Table S2.** Variations of top 10 genera along duodenum to colon between DL and HC groups.

**Table S3a.** Differential bacterial species between two groups in duodunum.

**Table S3b.** Differential bacterial species between two groups in jejunum.

**Table S3c.** Differential bacterial species between two groups in ileum.

**Table S3d.** Differential bacterial species between two groups in cecum.

**Table S3e.** Differential bacterial species between two groups in colon.

**Table S4a.** Species count in metagenomic WGCNA modules.

**Table S4b.** Detailed information of brown module in metagenomic modules.

**Table S4c.** Detailed information of grey module in metagenomic modules.

**Table S5a.** Discriminative KO genes between DL and HC groups in duodunum.

**Table S5b.** Discriminative KO genes between DL and HC groups in jejunum.

**Table S5c.** Discriminative KO genes between DL and HC groups in ileum.

**Table S5d.** Discriminative KO genes between DL and HC groups in cecum.

**Table S5e.** Discriminative KO genes between DL and HC groups in colon.

**Table S6a.** Key metabolic pathways related to microbial genes in duodunum.

**Table S6b.** Key metabolic pathways related to microbial genes in jejunum.

**Table S6c.** Key metabolic pathways related to microbial genes in ileum.

**Table S6d.** Key metabolic pathways related to microbial genes in cecum.

**Table S6e.** Key metabolic pathways related to microbial genes in colon.

**Table S6f.** The contribution of the most abundant genera to key metabolic pathways.
